# Vivo-Morpholino-Based Antiviral for SARS-CoV-2: Implications for Novel Therapies in the Treatment of Acute COVID-19 Disease

**DOI:** 10.3390/biomedicines9081018

**Published:** 2021-08-15

**Authors:** James E. K. Hildreth, Jon D. Moulton, Donald J. Alcendor

**Affiliations:** 1 Center for AIDS Health Disparities Research, Department of Microbiology and Immunology, School of Medicine, Meharry Medical College, 1005 Dr. D.B. Todd Jr. Blvd., Nashville, TN 37208, USA; jhildreth@mmc.edu; 2 Department of Internal Medicine, School of Medicine, Meharry Medical College, 1005 Dr. D.B. Todd Jr. Blvd., Nashville, TN 37208, USA; 3 Center for AIDS Health Disparities Research, Department of Microbiology, Immunology, and Physiology, School of Medicine, Meharry Medical College, 1005 Dr. D.B. Todd Jr. Blvd., Nashville, TN 37208, USA; 4 Gene Tools, LLC, 1001 Summerton Way, Philomath, OR 97370, USA; jmoulton@gene-tools.com

**Keywords:** coronavirus, SARS-CoV-2, COVID-19, dendrimers, morpholinos, cytotoxicity, MRCV-19

## Abstract

Therapeutic modalities designed specifically to inhibit COVID-19 infection and replication would limit progressive COVID-19-associated pulmonary disease in infected patients and prevent or limit systemic disease. If effective, antivirals could reduce viral transmission rates by reducing viral burden and allow time for immune clearance. For individuals infected with acute-stage disease, antivirals in support of the existing vaccines could reduce COVID-19 hospitalizations and deaths. Here, we evaluate MRCV-19, a phosphorodiamidate morpholino oligo with delivery dendrimer (Vivo-Morpholino), to prevent coronavirus infection in a cell culture model. This is a novel antiviral that effectively inhibits SARS-CoV-2 replication in vitro. By design, MRCV-19 targets the SARS-CoV-2 5’UTR and overlaps the *pp1a* start site of translation in order to block access of the translation initiation complex to the start. MRCV-19 testing is conducted in a high-throughput, 384-well plate format with a 10-point dose-response curve (common ratio of 2) assayed in duplicate with parallel cytotoxicity evaluations. MRCV-19 was shown to be more effective than hydroxychloroquine and remdesivir in our CPE reduction assay with low toxicity. The clinical translational impact of this study is providing the basis for evaluating MRCV-19 on a large scale in an appropriate infection model for toxicity and systemic high-level inhibition of SARS-CoV-2, which could lead in time to phase I testing in humans.

## 1. Introduction

Patients with pneumonias of unknown origin emerged in Wuhan City, Hubei Province, China, and were reported to the World Health Organization (WHO) on 31 December 2019 [1]. The WHO declared the outbreak an international public health emergency on 30 January 2020 [2]. Currently, the origin of COVID-19 is unknown, but speculation points to animal origin (zoonotic infection). COVID-19 is an emerging respiratory disease pathogen and currently the world’s greatest public health challenge, disrupting global economies [3]. With a fatality rate of ~1–2%, and the emergence of viral variants, it is projected that millions of people in the US and around the world will die of this disease [4]. Finding an efficacious therapeutic intervention in support of existing vaccines for individuals who have existing acute-stage disease is critical and most important for vulnerable populations, including the elderly and individuals with underlying medical conditions.

Coronaviruses (CoVs) are positive-sense, single-stranded, enveloped, RNA viruses that belong to the subfamily Coronavirinae, family Coronavirdiae, order Nidovirales. CoVs are classified into four genera: Alphacoronavirus (αCoV), Betacoronavirus (β-CoV), Deltacoronavirus (δCoV), and Gammacoronavirus (γCoV) [4]. To date, seven human coronaviruses (HCoVs) have been identified, two αCoVs (HCoV-229E and HCoV-NL63) and five β-CoVs (HCoV-OC43, HCoV-HKU1, severe acute respiratory syndrome-associated CoV (SARS-CoV), Middle East respiratory syndrome CoV (MERS-CoV), and most recently, SARS-CoV-2 (COVID-19)) [5,6,7,8]. The WHO has classified COVID-19 as a β-CoV of group 2B [9]. CoVs cause respiratory, enteric, hepatic, and neurological diseases in different animal species, including camels, cattle, cats, and bats [10]. The β-CoV lineages HCoV-OC43 and HCoV-HKU1 are usually associated with self-limiting upper respiratory infections in immunocompetent hosts and occasionally lower respiratory tract infections in immunocompromised hosts and the elderly [10]. Coronaviruses possess the largest genomes of all RNA viruses, consisting of about 30 kb of genomes. COVID-19 belongs to the β-CoV genera and has 89% nucleotide identity with bat-SARS-like (SL)-CoVZXC21 and 82% nucleotide identity with human SARS-CoV [11]. Examination of the viral evolution reveals bats and rodents are gene sources for most αCoVs and β-CoVs, while avian species are the proposed gene sources of most δCoVs and γCoVs [12]. CoVs often cross species barriers to infect humans and have emerged to cause significant morbidity and mortality in the general population. The most recent examples are SARS-CoV, emerging in China in 2002, with 8000 infections and 800 deaths [13,14], and the Middle East Respiratory Syndrome CoV (MERS-CoV), emerging in the Arabian Peninsula in 2012 [15,16,17]. Developing an antiviral for COVID-19 requires consideration of its genetic complexities and the potential to mutate in humans or a zoonotic intermediate host.

Here we utilize specific morpholino oligomers designed to block translation of the SARS-CoV-2 coronavirus RNA dependent RNA polymerase (RDRP) by binding to the 5′UTR of the pp1a subunit of the RDRP. Our antisense morpholino oligonucleotides are design to accomplish translation arrest of the virus RDRP by binding to and blocking the translational start site of mRNA molecules. The design of morpholino oligos can overcome many of the limitations of regular DNA oligos. Morpholinos designed by Summerton et al., in 1997 involved a DNA analog that acts by blocking translation [18]. The riboside moiety of each subunit is converted to a morpholine moiety (morpholine C4H9NO) and uses a phosphorodiamidate intersubunit linkage instead of phosphorodiester linkages. This allow the morpholino oligomer to only block translation when they are designed to be complementary to the 5′ leader sequences or to the first 25 bases 3′ to the AUG translational start site. Morpholinos act by preventing ribosomes from binding and they have greater efficiency and specificity than other antisense oligos because the 5′UTR is less conserved than coding regions and there is less nonspecific binding to wrong mRNAs. Here we demonstrate the ability of morpholino oligomers to inhibit the viral RDRP and protect cells from infection by SAR-CoV-2 in an infection assay.

## 2. Materials and Methods

### 2.1. Development of MRCV-1

Briefly, subunits are prepared from ribonucleosides (with the U base methylated to form T) by oxidizing open the ribose ring and reforming the ring on an ammonia, tritylating the morpholine nitrogen, adding appropriate protecting groups to the bases, and activating with dichloro dimethylamine phosphate. Oligos are assembled on a solid-phase resin, detritylating the new terminal morpholine ring before each round of subunit addition [18]. After oligo assembly, the activated dendrimeric precursor is added to the terminal morpholine nitrogen of the oligo while still on the oligo synthesis resin. This precursor has a primary amine at the terminus of each of the eight branches. Ammonia is added to cleave the oligo-dendrimer conjugate from the resin and to deprotect the bases. Next O-methyl isourea is added to guanidinylate the tips of the dendrimer. The product is isolated on solid-phase extraction columns then eluted. The Vivo-Morpholinos undergo MALDI-TOF analysis, UV quantification, aliquoting, freeze-drying and gentle baking [19]. Additional details of the synthesis and conjugation are proprietary

### 2.2. Assay Methods for Testing MRCV-19

#### 2.2.1. Screening Strategy

We employed a cell-based assay measuring the cytopathic effect (CPE) of the virus infecting Vero E6 host cells. The CPE reduction assay is popular and widely used to screen for antiviral agents due to its availability in high-throughput screening format [20,21]. In this assay, host cells infected with virus die as a consequence of the virus hijacking the cellular mechanisms for genome replication. The CPE reduction assay indirectly monitors the effect of antiviral agents acting through various molecular mechanisms by measuring the viability of host cells three days after virus inoculation. Antiviral compounds are identified as those protecting the host cells from the CPE of the virus, thereby increasing viability.

#### 2.2.2. Compound Preparation

Compound stock solutions (80 µL of 1.0 mM solution in water) were transferred into wells of an empty ECHO plate (stock plate). Compounds were diluted 2-fold by transferring 40 µL of each stock sample into an adjacent well containing 40 µL water and mixing. This process was repeated to create eight additional wells of serially diluted sample, each well containing a 2-fold diluted sample of the previous well. A 150 nL aliquot of each diluted sample of compounds GT1-18 and a 35 nL aliquot of each diluted sample for compounds MRCV-19 was dispensed into corresponding wells of assay-ready plates using an ECHO555 acoustic liquid handling system. The final assay concentration range was 5.0 µM–0.01 µM for compounds GT1-18 and 1.2 µM–0.002 µM for compounds MRCV-19.

### 2.3. Method for Measuring Antiviral Effect of Compounds

Vero E6 cells selected for expression of the SARS-CoV receptor (ACE2; angiotensin-converting enzyme 2) are used for the CPE assay [20]. Cells were grown in MEM supplemented with 10% HI FBS and harvested in MEM, 1% Pen/Strep, 1% HEPES, supplemented 2% HI FBS on the day of assay. Assay-ready plates pre-drugged with test compounds were prepared in the BSL-2 lab by adding 5 μL assay media to each well. The plates and cells were then passed into the BSL-3 facility. Cells were batch-inoculated with SARS-CoV-2 (USA_WA1/2020; M.O.I. ~ 0.002), resulting in 5% viability of untreated cells 72 h post-infection. A 25 μL aliquot of virus-inoculated cells (4000 Vero E6 cells/well) was added to each well in columns 3–24 of the assay plates. The wells in columns 23–24 contained only virus-infected cells for the 0% CPE reduction controls. Prior to virus inoculation, a 25 μL aliquot of cells was added to columns 1–2 of each plate for the cell-only 100% CPE reduction controls. After incubating plates at 37 °C/5% CO_2_ and 90% humidity for 72 h, 30 μL of Cell Titer-Glo (Promega Corporation, Madison, WI, USA) was added to each well. Plates were sealed with a clear cover, surface decontaminated, incubated at room temperature for 10 min, and then luminescence was read using a BMG CLARIOstar plate reader (BMG LABTECH Inc., Cary, NC, USA) to measure cell viability.

### 2.4. Method for Measuring Cytotoxic Effect of Compounds

Compound cytotoxicity was assessed in a BSL-2 counter screen as follows: Host cells in media were added in 25 μL aliquots (4000 cells/well) to each well of assay-ready plates prepared with test compounds as above. Cells only (100% viability) and cells treated with hyamine at 100 µM final concentration (0% viability) serve as the high- and low-signal controls, respectively, for cytotoxic effect in the assay. DMSO was maintained at a constant concentration for all wells as dictated by the dilution factor of stock test compound concentrations. After incubating plates at 37 °C/5% CO_2_ and 90% humidity for 72 h, 30 μL Cell Titer-Glo (Promega) was added to each well. After incubation at room temperature for 10 min, luminescence was read using a BMG PHERAstar plate reader (BMG LABTECH) to measure cell viability.

## 3. Data Analysis

For all assays, the raw data from plate readers were imported into ActivityBase (IDBS, Boston, MA, USA), where values were associated with compound IDs and test concentrations. For the antiviral CPE reduction assay, raw signal values were converted to % CPE reduction by the following formula: % CPE reduction = 100 × (test cmpd value − mean value infected cell controls)/(mean value uninfected cell controls − mean value infected cell controls). For the cell viability assay measuring compound cytotoxicity, % cell viability was calculated as follows: % viability = 100 × (test cmpd value − mean low-signal control)/(mean high-signal control − mean low-signal control). EC50 and CC50 values were calculated from a four-parameter logistic fit of data using the ActivityBase Xlfit module (IDBS).

## 4. Results

### 4.1. Strategy for MRCV-19 Inhibition of SARS-CoV-2 Replication

**Figure 1:**
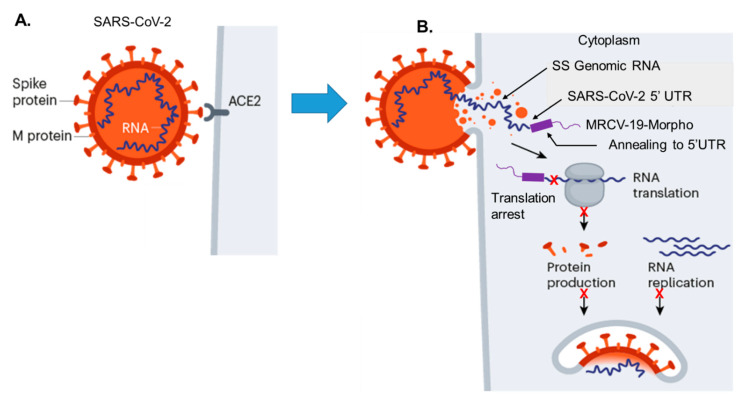
A model for MRCV-19 morpholino inhibition of SARS-CoV-2 protein expression translation arrest. (**A**) SARS-CoV-2 binding to the ACE2 receptor via the spike glycoprotein on permissive cells to initiate infection. (**B**) The release of single-stranded viral genomic RNA, and sequence-specific binding of MRCV-19 (GT4) to the 5′UTR of the *pp1a* gene loci to inhibit viral protein translation.

We show a hypothetical model of the MRCV-19 Morpholino inhibition of SARS-CoV-2 protein expression by translation initiation complex arrest at the 5′UTR just upstream of the *pp1a* start site of translation (Figure 1). After initial binding of the SARS-CoV-2 spike protein to the ACE2 receptor on permissive cells (Figure 1A), the viral genomic RNA gains access to the cell cytoplasm via membrane-mediated endocytosis (Figure 1B). After treatment of permissive cells with MRCV-19, the sequence-specific Morpholino immediately binds to the 5′UTR and overlaps the pp1a translation start site of the gene encoding the viral RNA-dependent RNA polymerase (RD-RNAP) (Figure 1B). In vivo delivery of MRCV-19 is accomplished via its octaguanidinium dendrimer group. MRCV-19 anneals to 5′UTR and binds across the *pp1a* start site (Figure 1B). The resulting hetroduplex cannot be translated by host cell polyribosomes. The overall effect is translational arrest of the RD-RNAP, which would block downstream protein expression efficiently.

### 4.2. Sequence Alignments and Morpholino Targeting in the 5′UTR

**Figure 2:**
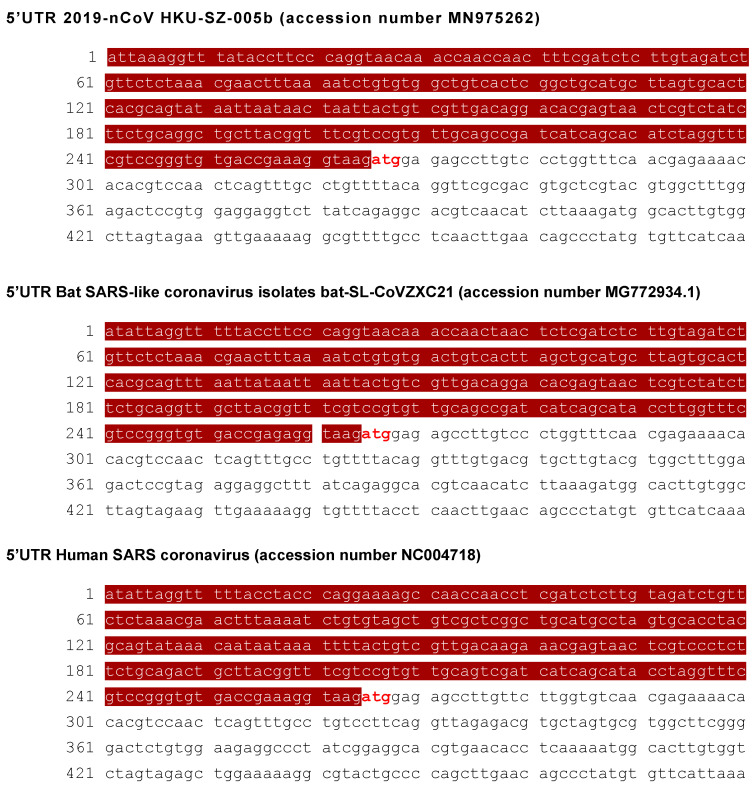
Sequence comparative alignment among SARS-CoV-2, bat-SL-CoVZXC21, and SARS-CoV-1 highlighting the 5′UTR (brown) and transcriptional start atg site of *pp1a* gene loci (red and white).

Selection of the 5′UTR target sequence was based on conserved sequences among SARS-CoV, SARS-CoV-2 (COVID-19), and bat-SL-CoVZXC21. These represent both human and animal sequences most similar to SARS-CoV-2, which causes COVID-19 [22,23,24]. The start site for *pp1a* (ATG) is demonstrated using orange blocks and white text (Figure 2). These include the 5′UTR pp1a genes sequences 5′UTR 2019-nCoV HKU-SZ-005 (accession number MN975262), 5′UTRBat SARS-like coronavirus isolate bat-SL-CoVZXC21 (accession number MG772934.1), and, 5′UTR human SARS coronavirus (accession number NC004718).

### 4.3. SARS-CoV-2 Antiviral (MRCV-19)

**Figure 3:**
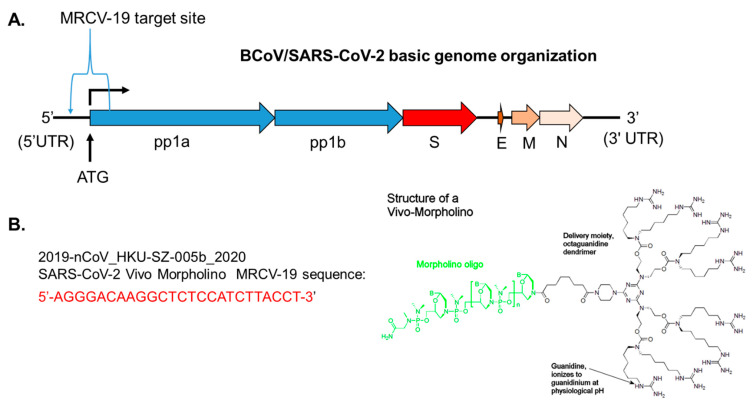
β-CoV/SARS-CoV-2 basic genome organization. (**A**) Genome organization of β-CoV. (**B**) Schematic structure of a Vivo-Morpholino composed of a 25-mer long morpholino oligonucleotide (green) covalently linked to an octaguanidine dendrimer (black), which serves as a delivery moiety. A nucleotide sequence of SARS-CoV-2 (2019-nCoV_HKU-SZ-005b_2020 Vivo-Morpholino MRCV-19) that is complementary to SARS-CoV-2 target sequence are shown in red text.

The SARS-CoV-2-targeted MRCV-19, a phosphorodiamidate Morpholino with delivery dendrimer (Vivo-Morpholino), is designed to be complementary to the 25-mer nucleotide sequence spanning the start of coding of the pp1a gene sequence, including the pp1a ATG codon designated as the MRCV-19 start site (Figure 3A). A sequence overlapping the 5′UTR and regions immediately downstream of the ATG start site was identified as the target to engage SARS-CoV-2 upon entry into permissive cells in vitro and in vivo based on the viral genome structure (Figure 3A). The structure of the Vivo-Morpholino and the complementary sequence targeting the SARS-CoV-2 genome are shown in Figure 3B.

The control dendrimer reagent used in this study was a standard control oligo targeting a human β-globin intron mutation that causes β-thalassemia. The standard control causes little change in phenotype in any known test system, except human β-thalassemic hematopoietic cells, and is an appropriate negative control for the custom Vivo-Morpholino.

### 4.4. MRCV-19 Inhibition of SARS-CoV-2 Infection

**Figure 4:**
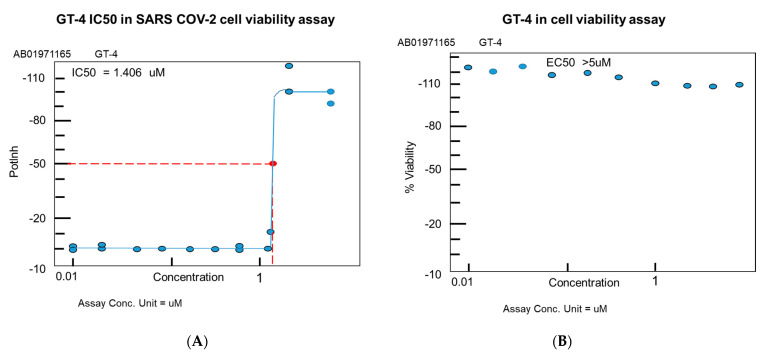
MRCV-19 inhibition of SARS-CoV-2 infection. MRCV-19 inhibition of SARS-CoV-2 infection in a luminescent cell viability assay. (**A**) IC50 values for MRCV-19. (**B**) Measure of MRCV-19 toxicity via a cell viability assay with no virus infection. Testing was conducted in a high-throughput, 384-well plate format with a 10-point dose response curve (common ratio of 2) assayed in duplicate with parallel cytotoxicity evaluation.

Cell viability, as measured by luminescence, showed an IC50 equal to 1.4 µM with a status active against SARS-CoV-2 infection, max inhibition of 115.58%, concentration at max inhibition of 2.5 µM, using a 20-point screen from 10 nM–5 µM concentration of MRCV-19 (Figure 4).

### 4.5. MRCV Exhibits Low Toxicity In Vitro

In viability assays using MRCV-19 alone, we observed a high level of viability after cellular exposure to MRCV-19 at different concentrations (Figure 4). We observed low toxicity over 72 h post-exposure. Toxicity analysis showed max viability of 132.35%, concentration at min % viability (2.500 µM), concentration at max % viability (0.039 µM), in a 10-point screen. MRCV-19 exhibited an EC50 value of >5 µM in the absence of SARS-CoV-2 (Figure 4).

### 4.6. MRVC-19, More Effective Than Hydroxychloroquine and Remdesivir to Inhibit SARS-CoV-2 Infection In Vitro

**Figure 5:**
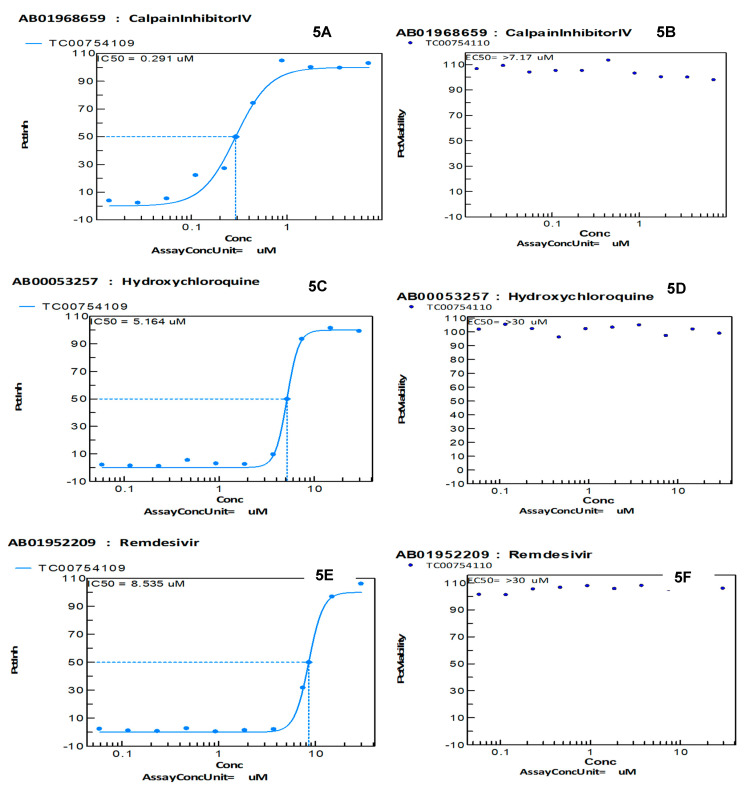
Calpain, hydroxychloroquine, and remdesivir inhibition of SARS-CoV-2 infection. (**A**) Calpain inhibition of SARS-CoV-2 infection in a luminescent cell viability assay with corresponding IC50 values. (**B**) Measure of calpain toxicity via a cell viability assay with no virus infection. (**C**) Hydroxychloroquine inhibition of SARS-CoV-2 infection in a luminescent cell viability assay with corresponding IC50 values. (**D**) Measure of hydroxychloroquine toxicity via a cell viability assay with no virus infection. (**E**) Remdesivir inhibition of SARS-CoV-2 infection in a luminescent cell viability assay with corresponding IC50 values. (**F**) Measure of remdesivir toxicity via a cell viability assay with no virus infection.

We compared the antiviral effects of calpain, hydroxychloroquine, and remdesivir for SARS-CoV-2 in our antiviral CPE reduction assay. We observed an IC50 value of 0.291 for calpain, which is known to produce high-level inhibition of SARS-CoV-2 in vitro (Figure 5A). Calpain alone in the absence of infection produced low-level toxicity with an EC50 > 7.17 µM in vitro (Figure 5B). Hydroxychloroquine produced an IC50 value of 5.164 in our CPE reduction assay after SARS-CoV-2 infection with low toxicity, having an EC50 value > 30 µM (Figure 5C,D). Finally, we examined remdesivir for virus inhibition and observed an IC50 value of 8.535 µM and low toxicity in the absence of virus infection with an EC50 > 30 µM. All toxicity assays were performed 72 h post-exposure.

## 5. Discussion

Development of an antiviral becomes appealing in support of vaccines. Targeting highly conserved sequences in both human and animal β-CoVs in the 5′ UTR would provide inhibition of SARS-CoV-2 replication, as well as existing β-CoVs (SARS-CoV, bat-SL-CoVZXC21), and likely future emerging SL-β-CoVs due to sequence conservation at the 5′UTR loci (Figure 2). Mutation accumulation at the pp1a locus, that encodes a portion of the RNA-dependent RNA polymerase, would be counterproductive to viral replication. We are aware that any level of viral protein expression could result in inflammation, which could impair immune function. Therefore, our strategy for developing MRCV-19 is to establish unique binding at the pp1a 5′UTR, inhibiting viral polymerase protein expression via translation arrest and so inhibiting all downstream viral protein expression. If successful, this strategy would be highly innovative, as our compound by design would be effective against future outbreaks of emerging SARS-β-CoVs, which share the highly conserved pp1a 5′UTR targeted by the MRCV-19 Morpholino antiviral.

MRCV-19 was shown to inhibit SARS-CoV-2 infection in vitro with low toxicity. MRCV-19 also was found to be more effective in inhibiting SARS-CoV-2 than hydroxychloroquine and remdesivir in our antiviral CPE reduction assay (Figure 4 and Figure 5). All four compounds tested showed low-level toxicity in our in vitro cell viability assays (Figure 4 and Figure 5). An important limitation of this study is that these findings may have no correlation with the in vivo effectiveness or toxicity of MRCV-19 compared to hydroxychloroquine and remdesivir. Our next steps involve in vivo testing in a Syrian hamster infection model for SARS-CoV-2 to determine efficacy and toxicity of MRCV-19 in vivo.

## 6. Conclusions

A COVID-19 vaccine would not be useful as an intervention for individuals with existing acute-stage disease that may become severe. However, an antiviral, such as MRVC-19 that is designed specifically for SARS-CoV-2, could be administered early during infection to reduce viral loads and provide the time needed for the immune system to recover and clear the virus in a timely manner, which would circumvent hospitalizations due to severe complication of COVID-19.

## Data Availability

The study did not report any laboratory-based data.
